# Supplementary material for: Reconciling Mining with the Conservation of Cave Biodiversity: A Quantitative Baseline to Help Establish Conservation Priorities
Source: PLoS One. 2016 Dec 20;11(12):e0168348. doi: 10.1371/journal.pone.0168348 (PMC5173368; doi:10.1371/journal.pone.0168348)
Supplement: S1 Dataset — (ZIP) [file pone.0168348.s002.zip › Taxa/Serra Norte/SN_2013/N4E/Lista N4E-54.pdf]

CAVIDADE N4E-0054

Classe	Ordem	Fam/Outros	Gên/Outros	Espécie	S-U	U-S	Zon.
Arachnida	Amblypygi	Phrynidae	*Heterophrynus*	*longicornis*	1		E
Arachnida	Araneae	Ctenidae		jovem	1		E
Arachnida	Araneae	Mysmenidae	*Microdipoena*	sp.1		X	E
Arachnida	Araneae	Ochyroceratidae	*Ochyrocera*	sp.1		X	E
Arachnida	Araneae	Pholcidae	*Leptopholcus*	sp.1	X		E
Arachnida	Araneae	Pholcidae	*Mesabolivar*	sp.1	X	X	E
Arachnida	Araneae	Theridiosomatidae	*Plato*	sp.1	X	X	E
Arachnida	Opiliones	Sclerosomatidae	*Prionostema*	sp.1	2		E
Arachnida	Pseudoscorpiones	Chernetidae	*Spelaeochernes*	sp.1	X	X	E
Entognatha	Collembola	Cyphoderidae		sp.1	X		E
Entognatha	Collembola	Sminthuroidea		sp.2	X		E
Entognatha	Diplura	Campodeidae		sp.1		X	E
Insecta	Coleoptera	Tenebrionidae		jovem		X	E
Insecta	Diptera	Chironomidae		sp.	X		E
Insecta	Diptera	Culicidae	*Culex*	sp.		X	E
Insecta	Diptera	Psychodidae	Phlebotominae	sp.	X	X	E
Insecta	Diptera	Tipulidae	Tipulinae	sp.	X		E
Insecta	Hymenoptera	Diapriidae		sp.1	X		E
Insecta	Hymenoptera	Formicidae	*Paratrechina*	sp.2		X	E
Insecta	Orthoptera	Phalangopsidae	*Paraclodes*	sp.	2	6	E
Insecta	Psocoptera	Epipsocidae	*Epipsocus*	sp.2	X		E
Amphibia	Anura	Leptodactylidae	*Leptodactylus*	cf. *vastus*		2	E
Amphibia	Anura	Strabomantidae	*Pristimantis*	cf. *fenestratus*	2		E
Mammalia	Chiroptera	Emballonuridae	*Peropteryx*	*kappleri*	2	2	E
Mammalia	Chiroptera	Phyllostomidae	*Carollia*	*perspicillata*	4		E
